# A Longitudinal Study of Changes in the Shot Characteristics of Women Table Tennis Players: Analysis of the Olympic Semifinals and Finals of Women's Singles

**DOI:** 10.3389/fpsyg.2022.901755

**Published:** 2022-06-30

**Authors:** Jie Wang, Mengqi Li, Xi Xiong

**Affiliations:** Key Laboratory of Diagnosis and Analysis of Skills and Tactics in Sports, College of Physical Education and Sports Training, Shanghai University of Sport, Shanghai, China

**Keywords:** table tennis, shot characteristics, longitudinal study, women's competition, notational analysis

## Abstract

This study aims to evaluate the changes in shot characteristics of elite women table tennis players through the longitudinal analysis of women's singles finals and semifinals from 2004 to 2021 Olympic Games. A total of 13 games were selected, and the stroke position, stroke type, ball placement, and stroke efficacy of 5,877 shots were analyzed using the notational analysis method. A chi-square test was used to test whether the shot characteristics had changed between game years, and the adjusted residual was calculated to judge these changes. In the four dimensions, namely, stroke position, stroke type, ball placement, and stroke efficacy, the shot characteristics of women table tennis players have changed with the year of the Olympic Games. The backhand stroke position has an upward trend from 55.0 to 58.1%, and the type of flip stroke has a downward trend from 7.6 to 2.1%. The ball placements of forehand and middle long have two fluctuations first rising from 11.2 to 12.7% and 20.3 to 23.7% and then falling from 12.7 to 5.3% and 23.7 to 16.0%. Moreover, backhand long is being increasingly used. In the year of equipment change, the proportion of neutral stroke increases from 40.0 to 61.5%. Once the players have become adapted to the equipment, the proportion of neutral stroke decreases from 61.5 to 48.5%. The development trend of women table tennis players is that the extreme stroke will be further increasingly used. Results suggest that elite women table tennis coaches and players should focus on the technical training in the forehand and backhand positions, strengthen the practice of topspin and block, and pay attention to the backhand long landing area. Women's table tennis coaches and players also need to pay close attention to the possible impact of changes in rules and equipment and carry out targeted training in advance to minimize the impact on the stroke efficacy of players.

## Introduction

Women's table tennis is an important part of all kinds of competitions. There are 2.5 possible gold medals (1 for singles, 1 for team, and 0.5 for mixed double events) for women's table tennis in the Olympic Games. Research on the competition of high-level women table tennis players can be helpful to clarify the development direction of this sport. They can also provide reference for the training and competition of women table tennis players. Table tennis competition is one of the fastest games globally, characterized by its endurance, intensive effort, and short duration (Kondrič et al., 2013; Zagatto et al., 2016). Whether a table tennis player can win a competition or not is restricted by many factors, such as physical fitness (Zagatto et al., 2010; Malagoli Lanzoni et al., 2014), psychological state, technical and tactical abilities (Munivrana et al., 2015), and environmental factors (Zagatto et al., 2010; Malagoli Lanzoni et al., 2014).

The technical and tactical ability of players often determines the outcome of the game (Yu et al., 2009; Malagoli Lanzoni et al., 2014; Munivrana et al., 2015). The technical and tactical ability of table tennis players is reflected in the shot characteristics, mainly including the stroke type, stroke position, stroke efficacy, and landing area of the ball. A notational analysis is a convenient and important approach to carrying out technical and tactical analysis by observation (Hughes and Bartlett, 2002; Hughes and Franks, 2004; Malagoli Lanzoni et al., 2014).

Research on shot characteristics of women table tennis players is mainly through case studies. Hot spots of these case studies are generally Chinese and Japanese players. In these studies, researchers mainly analyze competitions through the three-phase evaluation method (Wu and Li, 1992; Zhang et al., 2018; Zhao, 2019; Zhou et al., 2019; Chen, 2021; Zhang, 2021). The indicators for analysis of shot characteristics in women table tennis include stroke techniques (Zhang P. W., 2019), trajectory of the ball (Yu et al., 2008; Zhang P. W., 2019), landing area of the ball (Zhang, 2018; Zhang P. W., 2019), and tactics (Rong and Zhang, 2015; Lin and Li, 2017; Luo, 2018; Wang and Li, 2020). Case studies are meaningful to understand personalized characteristics of women table tennis players and significant to help them prepare for future trainings and competitions. However, it is difficult to draw conclusions that can be generalized.

Another frequently employed approach to research in this area is comparative studies. Comparative studies aim to determine the differences in shot characteristics between women players and teams. Research usually focuses on contrastive analyses between Chinese players or between Chinese and Japanese players (Jia, 2013; Zhu, 2015; Luo, 2016; Yan, 2016; Liu, 2018; Ma, 2018; Jiang, 2019; Xu, 2019; Lu, 2020). Most of these studies analyze competitions according to the three-phase evaluation method. In these studies, competitions are often divided into three phases, namely, the serve-attack phase, return-attack phase, and stalemate phase. In serve-attack phases, researchers tend to focus more on analyses of the serving types, serving positions, rotations of the ball and techniques of the third stroke. In return-attack phases, researchers are more concerned with stroke types of the second stroke, positions of reserving, the convergence between the second and fourth strokes and the landing area of the fourth stroke. In stalemate phases, tactical combinations and tactical situation are often the discussion point in some of these studies. Tactical combinations in these studies often include the combination of stroke positions and stroke techniques (Zhang, 2021), that of stroke techniques and landing areas of the ball, and the combination of stroke types and the trajectories of the ball as well as other types of combinations. The table tennis game is a game with a process full of dynamic interactions between both sides of the game (McGarry et al., 2002; Glazier, 2010; Marcelino et al., 2011; Gómez et al., 2017). Thus, tactical situations (strategies) are often used to indicate the contrast of strength between the two sides in a game. Tactical situations in stalemate phases include the active state, passive state, and even state (Chen, 2021). In different tactical situations, players may perform various techniques and show different technical behaviors. Therefore, comparative studies were meaningful to determine the handling of high-level players and provide references for other players. However, the conclusions were still cross-sectional, such as those in case studies.

Longitudinal studies are useful to find out changes in shot characteristics over a long period and predict the future development of techniques and tactics employed in table tennis competitions. A few longitudinal studies investigate the characteristics of women's shots in table tennis matches. However, these studies mainly focus on changes in 2 or 3 years' period of times. Zhang R. J. (2019) analyzed, in 30 games selected from 2014 to 2016, changes in technical and tactical applications of Ding Ning, Liu Shiwen, and Zhu Yuling of Chinese women table tennis players and provided suggestions for their follow-up trainings and competitions. Chen (2013) selected 20 games of Ding Ning from 2010 to 2012, analyzed the games using the three-phase method, and found some rules of Ding's skills and tactics. It can be seen that the above studies mainly focused on certain skills or tactics of certain players and proposed individualized advice for players, mostly within the time span of no more than 3 years. That would be very difficult in understanding the evolution process of shot characteristics. Considering the limited samples of their research subjects, it was very difficult to draw general conclusions from their research findings. In view of the limitations of existing studies, this study, through the notational analysis of all the Olympic finals and semifinals since 2004, aims to analyze the changes in table tennis in women's singles, the changes in high-level women table tennis players in their strokes, and to summarize the general laws of these changes. It also attempts to speculate the future trend and characteristics of shot development so as to provide support for decision of high-level women table tennis players in their preparations for and participations in the competitions. The assumption is that shot characteristics in high-level women table tennis competitions have changed along with the Olympic years since 2004.

## Methods

### Sample Selection

Competitions were selected from the finals and semifinals of women table tennis singles in the Olympic Games from 2004 to 2021. For the particularity of the chopping playing style, those competitions with choppers were excluded from the analysis. Finally, 13 competitions were chosen, with 26 player-games. A total of 5,877 strokes were included for analysis. The videos of these competitions were downloaded from bilibili.com or v.qq.com. This study was approved by the Academic Committee of the School of Physical Education and Sports Training, Shanghai University of Sport.

### Analysis Indicators

Following previous studies (Yu et al., 2008), stroke position, stroke type, stroke efficacy, and ball placement of every shot were analyzed with the exclusion of serve for its unique and technical particularity. The definitions of the other four expressions are as follows: (1) stroke position is the body posture of a player, including four types of position, namely, forehand, backhand, pivot, and backhand turn position (Yu et al., 2008; Wang J., 2019). (2) Stroke type is the action adopted by a player when hitting the ball, which can be divided into six types, namely, topspin, flip, chop, chopping short, block, and others (Zhang et al., 2010; Malagoli Lanzoni et al., 2013, 2014; Wang J., 2019). (3) Stroke efficacy is the evaluation of the stroke effect of a player, which can be defined into five types, namely, very good, good, neutral, poor, and very poor (Malagoli Lanzoni et al., 2011a,b; Wang J., 2019). Finally, (4) ball placement is the landing area, where the ball falls on the table after a player hits the ball. It can be divided into nine types, namely, forehand short, forehand half, forehand long, middle short, middle half, middle long, backhand short, backhand half, and backhand long (Zhang et al., 2010; Malagoli Lanzoni et al., 2014; Wang J., 2019).

### Data Collection

A human–computer interaction observational tool was developed for notational analysis, in which one stroke was taken as an observation unit and the information of every stroke was recorded, including stroke position, stroke type, stroke placement, and stroke efficacy of every stroke. Serve was excluded from analysis for its technical particularity. Two observers participated in the data collection. One had more than 15 years of experience in the technical and tactical analysis of table tennis, and the other was the level A table tennis player of the Chinese Table Tennis Association. First, the two observers received training on observation indicators, observation tools, and observation process. Then, one match was randomly selected and assigned to two observers for independent analysis and data collection. A week later, one of the observers repeated observation and data collection of the match. The intergroup and intragroup observer reliabilities were calculated through a kappa coefficient. The target value of intergroup and intragroup reliability of each observation index was set to 0.85. When the reliability calculated by observation did not meet the target value, the two observers would discuss the inconsistent observation results to reach a consensus.

### Statistical Analysis

Based on the purpose of the study, a chi-square test for independence was used to analyze the data (Laffaye et al., 2015). The null hypothesis was that the percentages of stroke position, stroke type, ball placement, and stroke efficacy used in the Olympic Games remained similar with the years of Olympic Games. The alternative hypothesis was that the percentages of stroke position, stroke type, ball placement, and stroke efficacy used in the Olympic Games had changed with the years of Olympic Games. Statistical significance was set at *p* < 0.05. Once the difference between shot characteristics and Olympic Games year was found, a *post-hoc* test would be conducted to determine the differences among the groups. The adjusted residual was calculated to determine the difference among groups. When the adjusted residual was >3 or lower than −3, we believe that the difference between the observed value and the expected value of the index was statistically significant (Glen, 2013). All statistical analyses were performed using SPSS 24.0.

## Results

The percentages of stroke position (χ^2^ = 62.500; *p* < 0.010), stroke type (χ^2^ = 172.274; *p* < 0.010), ball placement (χ^2^ = 117.080; *p* < 0.010), and stroke efficacy (χ^2^ = 125.493; *p* < 0.010) have changed with the year of the Olympic Games. Tables 1–4 present the counts and percentages of each variable related to shot characteristics throughout the different Olympic Games from 2004 to 2021.

**Table 1:** The distribution of stroke position throughout the different Olympic Games from 2004 to 2021.

		**OG-04**	**OG-08**	**OG-12**	**OG-16**	**OG-21**	**Total**
Forehand	Count	214	401	548	252	322	1,737
	Expected count	202.5	435.1	514.0	248.0	337.5	1,737.0
	% of stroke position	31.2%	27.2%	31.5%	30.0%	28.2%	29.6%
	Adjusted residual	1.0	−2.2	2.1	0.3	−1.1	
Backhand	Count	377	773	852	413	663	3,078
	Expected count	358.8	770.9	910.8	439.4	598.1	3,078.0
	% of stroke position	55.0%	52.5%	49.0%	49.2%	58.1%	52.4%
	Adjusted residual	1.5	0.1	−3.4^*^	−2.0	4.3^*^	
Pivot	Count	76	233	293	149	142	893
	Expected count	104.1	223.7	264.2	127.5	173.5	893.0
	% of stroke position	11.1%	15.8%	16.8%	17.8%	12.4%	15.2%
	Adjusted residual	−3.2^*^	0.8	2.3	2.2	−2.9	
Backhand turn	Count	18	65	46	25	15	169
	Expected count	19.7	42.3	50.0	24.1	32.8	169.0
	% of stroke position	2.6%	4.4%	2.6%	3.0%	1.3%	2.9%
	Adjusted residual	−0.4	4.1^*^	−0.7	0.2	−3.5^*^	
Total	Count	685	1,472	1,739	839	1,142	5,877
	Expected count	685.0	1,472.0	1,739.0	839.0	1,142.0	5,877.0
	% of stroke position	100.0%	100.0%	100.0%	100.0%	100.0%	100.0%

* *The significant difference between the observed value and the expected value of the index*.

**Table 2:** The distribution of stroke type throughout the different Olympic Games from 2004 to 2021.

		**OG-04**	**OG-08**	**OG-12**	**OG-16**	**OG-21**	**Total**
Topspin	Count	360	924	1,088	540	714	3,626
	Expected count	422.6	908.2	1,072.9	517.6	704.6	3,626.0
	% of year	52.6%	62.8%	62.6%	64.4%	62.5%	61.7%
	Adjusted residual	−5.2^*^	1.0	0.9	1.7	0.6	
Flip	Count	52	91	54	30	24	251
	Expected count	29.3	62.9	74.3	35.8	48.8	251.0
	% of year	7.6%	6.2%	3.1%	3.6%	2.1%	4.3%
	Adjusted residual	4.6^*^	4.2^*^	−2.9	−1.1	−4.0^*^	
Chop	Count	101	96	113	60	135	505
	Expected count	58.9	126.5	149.4	72.1	98.1	505.0
	% of year	14.7%	6.5%	6.5%	7.2%	11.8%	8.6%
	Adjusted residual	6.1^*^	−3.3^*^	−3.7^*^	−1.6	4.3^*^	
Chopping short	Count	35	84	123	55	69	366
	Expected count	42.7	91.7	108.3	52.3	71.1	366.0
	% of year	5.1%	5.7%	7.1%	6.6%	6.0%	6.2%
	Adjusted residual	−1.3	−1.0	1.7	0.4	−0.3	
Block	Count	130	272	304	139	180	1,025
	Expected count	119.5	256.7	303.3	146.3	199.2	1,025.0
	% of year	19.0%	18.5%	17.5%	16.6%	15.8%	17.4%
	Adjusted residual	1.1	1.2	0.1	−0.7	−1.7	
Others	Count	7	5	57	15	20	104
	Expected count	12.1	26.0	30.8	14.8	20.2	104.0
	% of year	1.0%	0.3%	3.3%	1.8%	1.8%	1.8%
	Adjusted residual	−1.6	−4.8^*^	5.7^*^	0.0	−0.1	
Total	Count	685	1,472	1,739	839	1,142	5,877
	Expected count	685.0	1,472.0	1,739.0	839.0	1,142.0	5,877.0
	% of year	100.0%	100.0%	100.0%	100.0%	100.0%	100.0%

* *The significant difference between the observed value and the expected value of the index*.

**Table 3:** The ball placement throughout the different Olympic Games from 2004 to 2021.

		**OG-04**	**OG-08**	**OG-12**	**OG-16**	**OG-21**	**Total**
Forehand short	Count	6	11	14	5	9	45
	Expected count	4.8	11.4	13.9	6.2	8.7	45.0
	% of year	1.2%	0.9%	1.0%	0.8%	1.0%	1.0%
	Adjusted residual	0.6	−0.1	0.0	−0.5	0.1	
Forehand half	Count	46	95	123	51	49	364
	Expected count	38.9	91.9	112.4	50.5	70.2	364.0
	% of year	9.2%	8.0%	8.5%	7.8%	5.4%	7.8%
	Adjusted residual	1.3	0.4	1.2	0.1	−2.9	
Forehand long	Count	56	151	118	68	48	441
	Expected count	47.2	111.4	136.2	61.2	85.0	441.0
	% of year	11.2%	12.7%	8.1%	10.4%	5.3%	9.4%
	Adjusted residual	1.4	4.6^*^	−2.0	1.0	−4.7^*^	
Middle short	Count	12	50	62	25	34	183
	Expected count	19.6	46.2	56.5	25.4	35.3	183.0
	% of year	2.4%	4.2%	4.3%	3.8%	3.8%	3.9%
	Adjusted residual	−1.8	0.7	0.9	−0.1	−0.2	
Middle half	Count	91	270	371	184	219	1,135
	Expected count	121.4	286.7	350.5	157.6	218.8	1,135.0
	% of year	18.1%	22.8%	25.6%	28.2%	24.2%	24.2%
	Adjusted residual	−3.3^*^	−1.3	1.5	2.6	0.0	
Middle long	Count	102	281	284	104	186	957
	Expected count	102.3	241.7	295.6	132.9	184.5	957.0
	% of year	20.3%	23.7%	19.6%	16.0%	20.6%	20.4%
	Adjusted residual	0.0	3.3^*^	−0.9	−3.0^*^	0.1	
Backhand short	Count	3	6	8	5	5	27
	Expected count	2.9	6.8	8.3	3.7	5.2	27.0
	% of year	0.6%	0.5%	0.6%	0.8%	0.6%	0.6%
	Adjusted residual	0.1	−0.4	−0.1	0.7	−0.1	
Backhand half	Count	72	115	229	92	140	648
	Expected count	69.3	163.7	200.1	90.0	124.9	648.0
	% of year	14.3%	9.7%	15.8%	14.1%	15.5%	13.8%
	Adjusted residual	0.4	−4.7^*^	2.6	0.2	1.6	
Backhand long	Count	114	207	241	118	215	895
	Expected count	95.7	226.1	276.4	124.3	172.5	895.0
	% of year	22.7%	17.5%	16.6%	18.1%	23.8%	19.1%
	Adjusted residual	2.2	−1.6	−2.8	−0.7	4.0^*^	
Total	Count	502	1,186	1,450	652	905	4,695
	Expected count	502.0	1,186.0	1,450.0	652.0	905.0	4,695.0
	% of year	100.0%	100.0%	100.0%	100.0%	100.0%	100.0%

* *The significant difference between the observed value and the expected value of the index*.

**Table 4:** The distribution of stroke efficacy throughout the different Olympic Games from 2004 to 2021.

		**OG-04**	**OG-08**	**OG-12**	**OG-16**	**OG-21**	**Total**
Very good	Count	159	266	273	172	205	1,075
	Expected count	125.3	269.3	318.1	153.5	208.9	1,075.0
	% of year	23.2%	18.1%	15.7%	20.5%	18.0%	18.3%
	Adjusted residual	3.5^*^	−0.3	−3.3^*^	1.8	−0.3	
Good	Count	31	50	39	22	46	188
	Expected count	21.9	47.1	55.6	26.8	36.5	188.0
	% of year	4.5%	3.4%	2.2%	2.6%	4.0%	3.2%
	Adjusted residual	2.1	0.5	−2.7	−1.0	1.8	
Neutral	Count	274	829	1,070	407	599	3,179
	Expected count	370.5	796.2	940.7	453.8	617.7	3,179.0
	% of year	40.0%	56.3%	61.5%	48.5%	52.5%	54.1%
	Adjusted residual	−7.9^*^	2.0	7.4^*^	−3.5^*^	−1.2	
Poor	Count	38	41	68	51	55	253
	Expected count	29.5	63.4	74.9	36.1	49.2	253.0
	% of year	5.5%	2.8%	3.9%	6.1%	4.8%	4.3%
	Adjusted residual	1.7	−3.3^*^	−1.0	2.7	0.9	
Very poor	Count	183	286	289	187	237	1,182
	Expected count	137.8	296.1	349.8	168.7	229.7	1,182.0
	% of year	26.7%	19.4%	16.6%	22.3%	20.8%	20.1%
	Adjusted residual	4.6^*^	−0.8	−4.3^*^	1.7	0.6	
Total	Count	685	1,472	1,739	839	1,142	5,877
	Expected count	685.0	1,472.0	1,739.0	839.0	1,142.0	5,877.0
	% of year	100.0%	100.0%	100.0%	100.0%	100.0%	100.0%

* *The significant difference between the observed value and the expected value of the index*.

For stroke position, the count of backhand in OG-12 was significantly lower than the expected count (adjusted residual < −3), and that of OG-21 was significantly higher than the expected count (adjusted residual > 3). The percentage of backhand decreased in 2012 and then increased in 2021, both significantly. For the pivot position, the count was significantly lower than the expected count (adjusted residual < −3). The backhand turn position showed a significant relationship between OG-08 (adjusted residual > 3) and OG-21 (adjusted residual < −3). The percentage of forehand position was stable at ~30%.

For stroke type, the count of topspin in OG-04 was significantly lower than the expected count (adjusted residual < −3). From 2008 to 2021, the proportion of topspin was stable at more than 60%. The count of flip had relatively frequent fluctuations, higher than the expected count in OG-04 (adjusted residual > 3) and OG-08 (adjusted residual > 3) and lower than the expected count in OG-21 (adjusted residual < −3). The count of chop had more frequent fluctuations in OG-04 (adjusted residual > 3), OG-08 (adjusted residual < −3), OG-12 (adjusted residual < −3), and OG-21(adjusted residual > 3). The distribution of chopping short and block types was relatively stable. The count of other stroke types had two fluctuations in OG-08 (adjusted residual = −4.8) and OG-12 (adjusted residual > 3).

For ball placement, the count of forehand long in OG-08 was significantly higher than the expected count (adjusted residual > 3) and significantly lower than the expected count in OG-21 (adjusted residual < −3). The count of the middle half was significantly lower than the expected count in OG-04 (adjusted residual < −3). The proportion of middle long had two changes in OG-08 (adjusted residual > 3) and OG-16 (adjusted residual < −3). The count of the backhand half was significantly lower than the expected count (adjusted residual < −3). The proportions of forehand short, forehand half, middle short, and backhand short remained_stable from 2004 to 2021. The count of backhand long was significantly higher than the expected count in OG-21.

For stroke efficacy, significant changes existed in very good, neutral, poor, and very poor types. Both very good and very poor types had two fluctuations in OG-04 and OG-12. The count of very good in OG-04 was significantly higher than the expected count (adjusted residual > 3) and lower than the expected count in OG-12 (adjusted residual < −3). The count of very poor in OG-04 was significantly higher than the expected count (adjusted residual > 3) and significantly lower than the expected count in OG-12 (adjusted residual < −3). The count of neutral had three fluctuations in 2004 (adjusted residual < −3), 2012 (adjusted residual > 3), and 2016 (adjusted residual < −3). The count of poor was lower than the expected count in 2008 (adjusted residual < −3).

## Discussion

As already mentioned before, this study aims to analyze the changes during the years 2004–2021 of elite women table tennis players in the characteristics of their shots. The results indicate that significant changes have happened in all the analyzed dimensions (i.e., stroke position, stroke type, ball placement, and stroke efficacy) since 2004.

### Changes in Stroke Position

Strength is one of the five elements essential to winning a table tennis game (Fang, 2004). Stroke position is the premise for table tennis players to exert their strength. Appropriate stroke position is conducive to the athletes' exerting of their strength and preparation for the execution of tactics (Wang J., 2021). Forehand and backhand positions are important stroke positions of women's table tennis players, and the average ratio is ~82% since 2004. In this study, the forehand position refers to the position when table tennis players stand in the forehand position and hit the ball with the forehand. The backhand position is that when they stand in the backhand position and hit the ball with the backhand. The pivot position is the position when the players stand in the backhand position and hit the ball with the forehand, while the backhand turn position refers to that when they stand in the forehand position and hit the ball with the backhand. Therefore, the forehand and backhand positions are comfortable hitting positions for players, whereas the pivot and backhand turn positions are not easy to implement. Therefore, the proportions of forehand and backhand positions are relatively high, whereas the proportions of pivot and backhand turn positions are relatively low. From 2004 to 2021, no significant changes are found in the percentage of forehand positions. However, the percentage of backhand shots significantly increased by 9.1%, and the percentage reached 58.1% in 2021, which is a very important change. According to the relevant literature, backhand is the preferred stroke position of men's table tennis players because the stroke position is not only easy to perform but may also cause return difficulties for opponents (Liu, 2011). In recent years, many national teams have implemented the strategy of masculinization of women's playing style, that is, women's table tennis players learn to adopt the playing method of their male counterparts (Bai, 2008). And the significant increase of backhand stroke in women's table tennis players may be related to the masculinization of women's playing style.

### Changes in Stroke Type

Stroke type includes six types, namely, topspin, flip, chop, chopping short, block, and others (Wang J., 2019). For attacking style players, topspin is the main offensive mean and accounts for ~60%. Block is the main defensive mean and accounts for ~17%, and chopping short is the main control mean and accounts for ~6%. The results showed that no significant change existed in the utilization rate of the above three types from 2008 to 2021. Only the utilization rate of topspin was significantly lower than the average value in 2004. Given its emergence, the topspin type has gradually broken the technical monopoly of backhand-block with forehand-attack and near-table fast attack because of its offensive stability and the ability to create strong rotation. This type has also developed into the most common offensive method used by modern table tennis players (Liu, 2010). In all statistical years, the usage of topspin has reached ~60%, accounting for more than half of all techniques, hence the most frequently used ones by women's table tennis players. Although women's players have always been using the masculine playing style, yet they have gained limited scores directly by offensive means in the first-three strokes, owing to their slower speed and weaker strength. Therefore, most players still choose the topspin type because of its higher stability and easier exertion in the attacking and stalemate stages.

The proportion of flip type was gradually declining from 2004 to 2021. In 2004, the stroke type of flip accounted for 7.6% of the use of all techniques. However, by 2021, the proportion had dropped to 2.1%. The changes may be related to the changes in the ball materials and those in women's playing styles. Before 2009, there had been no changes in the materials of table tennis balls, and the ball speed had been faster; therefore, the flip type posed a higher threat and remained a more effective scoring method. However, after the use of inorganic glue (2009) and large-diameter plastic balls (2014), the ball's rotation was reduced along with its speed, which would make it more difficult for the player to make a strong backspin. As a result, the flip type was gradually being replaced by other offensive types, and the usage rate of flip was on the decline and fell to 2.1% in 2021.

The proportion of chop shows relatively large fluctuations from 2004 to 2021. In 2004, the proportion of chop was very high and reached 14.7%. During this period, the women's style of play was relatively conservative, and they would not blindly choose to attack when dealing with a high-quality chop. Since 2004, women's style of skills has gradually become aggressive, and many athletes have a good attack ability when handling the chop. The choice of the chop may mean that the player may fall into a passive defensive stage. Therefore, the proportion of the chop in 2008 and 2012 was as low as 6.5%. After 2016, affected by changes in women's play style and table tennis rules, the proportion of chop has gradually increased and once reached 11.8% in 2021. First, with the masculine development of women's techniques and the use of plastic balls from 2014, the chop is no longer just an excessive technical means. Due to the reduction in speed and spin of the ball, the quality and threat of the topspin are also reduced. When the quality of the chop is high, it would be very difficult for the opponent to improve the quality of the attack. When the opponent makes a low-quality chop, counter loop would prove to be a very effective offensive method. The tactic of active chop and counter loop has been very common in men's games. However, women players have been increasingly using such techniques as their play style gradually approaches men. Second, with the increasing use of the backhand-twisting technique, players can no longer limit the opponent's active attacks simply by using the technique of chopping short. In this case, the active chop can be employed to effectively limit the moving range of the opponent's positions and prevent him in advance from standing near the table so as to control his short ball or attack.

The proportion of blocks used in women's games has been relatively stable but still showed a downward trend from 19 to 15.8%. This change is also in line with the development of women's play styles. Currently, women players are becoming increasingly aggressive in their styles of play; therefore, it becomes very difficult to be solely relying on defense or the opponent's mistakes for scoring. Therefore, fewer consecutive attacks and defenses are to be found in the games. Most athletes will look for opportunities to counterattack in defense and turn to the stalemate stage (Wang M. J., 2019).

### Changes in Ball Placement

Ball placement is affected by the power, angle, and position of a player when hitting the ball, and also influenced by the player's tactical considerations. The ball placement of the middle half accounts for a relatively high proportion in women's games, with an average proportion of ~24%. Except for 2004, the proportion of ball placement landing in this area was stable. The proportion in 2004 was significantly less than other years and accounted for only 18.1%. The ball placement at this landing area generally occurs when players are performing chopping-short shot and stalemate tactics. The chopping short to this landing area can effectively prevent the opponent from making a large-angle chop or a high-quality chopping short. In the tactical stalemate, women players were using less of sideways, and most players preferred the backhand quick-topspin technique. Therefore, by landing the ball at the middle position players can effectively limit the fast attack of the opponent's backhand attack. According to the results of analysis of stroke type and stroke efficacy, women players preferred to use flip and chop types in dealing with short balls in 2004, and fewer rounds entered the stalemate stage. Therefore, the middle half placement in 2004 was significantly less than other years.

The ball placement of forehand long and middle long accounted for ~30%. In 2008, women players chose more forehand long (12.7%) and middle long (23.7%) ball placement than other years. The landing area of the ball was related to the selections of stroke types and stroke positions. The players preferred to choose the backhand landing area of the ball when performing the chop type because of the limit of space and time. The decrease in the utilization rate of the chopping type (from 14.7 in 2004 to 6.5% in 2008) will inevitably lead to the change of the ball placement from the backhand area to the middle and forehand area.

In 2016, the ball placement of the middle long was significantly lower than other years and accounted for only 16%. This change may be related to the change of ball. According to the requirements of the International Table Tennis Federation, plastic balls have been used to replace celluloid balls in table tennis matches since 2014. The diameter of the new ball increased, the speed and rotation decreased compared with the previous ones (Xiao et al., 2019) and the landing placements of the ball became shorter. This decrease may also be related to the tactical consideration of players. With the implementation of the masculine strategy among women players, many of them have enhanced their offensive abilities. The players will seize all possible opportunities to attack, and the low-quality middle long ball placement is very likely to give opponents opportunity for counterattacks. Therefore, players will deliberately reduce the use of middle long ball placement.

In 2021, the landing area of forehand long had decreased to the point of 5.3%, whereas the landing area of backhand long had significantly increased to 23.8%. This change may also be caused by the masculinization playing style among women players. As women players are increasingly using the masculine strategies, their offensive abilities are also enhanced (Zhu, 2014). A forehand attack is the most effective means of attack. Therefore, players should try to use fewer forehand long ball placements so as not to leave any chances for opponents to attack. More use of backhand long ball placements may render it more difficult for the opponent to attack while creating more opportunities for his own counterattack. This change may also be related to the popularity of active chop techniques in women's competitions. The high-quality active chop can prevent the opponent from hitting the ball with high speed and strong rotation. Moreover, players preferred to deploy backhand long ball placement by chopping to increase the difficulty of their opponents. From the result of stroke type, the usage ratio of chop reached 11.8% in 2021, which was higher than that of previous years. The increase in the proportion of chopping may lead to an increase in the proportion of backhand long ball placement.

### Changes in Stroke Efficacy

In terms of stroke efficacy, the changes in the proportion of the very good and very poor are very similar. Their utilization rates were significantly higher than the multiyear average in 2004 and significantly lower than the multiyear average in 2012. This trend of changes in the utilization rate of neutral stroke was just the opposite. The utilization rate of neutral strokes was significantly lower than that of the multiyear average in 2004, whereas the utilization rate in 2012 was significantly higher than the multiyear average. In 2004, inorganic glue and large diameter plastic balls were not yet used in a table tennis competition, the speed of the ball was relatively fast, and the rotation was relatively strong. Players were likely to obtain more opportunities or scores by rotating changes in table tennis matches and to score a high-quality stroke with strong rotation through spin control and active acceleration. Moreover, given the strong rotation of the ball, it was difficult to control the ball. A judgment deviation or improper position would cause very poor stroke efficacy. In 2012, the proportion of the very good and very poor strokes dropped significantly to 15.7 and 16.6%. However, the proportion of neutral strokes increased significantly to 61.5%. This change may be related to the change of ball. Considering that the speed and spin of the ball were reduced after the use of inorganic glue, creating opportunities by accelerating and adding spin is more difficult for players (Li and Zhang, 2010). The transition from backspin to topspin was more likely for players to make mistakes. Therefore, players became conservative in their strokes and tended to use more transitional shots to create offensive opportunities. In this way, the proportion of neutral strokes would increase, whereas the proportion of very good and very poor strokes would decrease. By 2016, women players had gradually become adapted to the plastic ball, and the impact of the change of ball material and diameter on athletes had become less evident. Excellent women players were trying to use more offensive means to take the initiative. The proportion of neutral strokes had decreased significantly.

## Limitation

This study has its limitations. As a longitudinal study, this study does not include all the Olympic Games of women table tennis players. It only selects the games in 2004 and beyond for research. The main reason is that the videos before 2004 were not found or incomplete. The second limitation was that this study included the women table tennis players with offensive playstyle but not the players with the chopping play style. We hope to find earlier game videos in the future and carry out research with longer time span to present a clearer picture of the characteristics of women's table tennis players. In addition, further research into the stroke characteristics of women table tennis players with the chopping style can be carried out separately and hopefully some rules can be found to provide decision support for the training and competition of chopping style players.

## Conclusion

In the four dimensions of stroke position, stroke type, ball placement, and stroke efficacy, the shot characteristics of women table tennis players have changed with the year of the Olympic Games, hence refused the null hypothesis initially proposed, but confirmed the proposed alternative hypothesis. Forehand and backhand positions are the most important stroke positions of women table tennis players. The utilization rate of the forehand position has remained unchanged, and that of the backhand position had an upward trend. Topspin was the most important way of attack, and block was the most important way of defense. The utilization rate of these two techniques had not changed. Chop and chopping short were the major means of control. The utilization rate of chopping short had not changed, but that of the chop fluctuated frequently. The type of flip stroke had a downward trend. Middle half, middle long, and backhand long ball were the main ball placements of women table tennis players. The ball placements of middle long first increased and then decreased. The latest trend is the increase in the proportion of ball placement of backhand long. The change in stroke efficacy is closely related to the change in table tennis equipment. In the initial stage of equipment change, the proportion of neutral stroke increases. Once the players are adapted to the equipment, the proportion of extreme stroke efficacy increases. The development trend of women table tennis players is that the proportion of extreme stroke will further increase. The findings suggest that elite women table tennis players should focus on the technical training in forehand and backhand positions and highlight the practice of topspin and block. In the reserve training, women players should strengthen the reserving practice of the whole table and pay special attention to the backhand long ball placement. Women's table tennis coaches and players also need to pay close attention to the possible impact of changes in competition rules and equipment and carry out targeted training in advance to minimize the impact on the stroke efficacy of players.

## Data Availability Statement

The datasets presented in this article are readily available upon request to JW 153627313@qq.com.

## Ethics Statement

This study was approved by the College of Physical Education and Sports Training Academic Committee, Shanghai University of Sport. Written informed consent for participation was not required for this study in accordance with the national legislation and the institution requirements.

## Author Contributions

JW: conceptualization, methodology, writing, and funding acquisition. ML: data collection. XX: literature search. All authors contributed to the article and approved the submitted version.

## Funding

This study was supported by the Shanghai Key Lab of Human Performance (Shanghai University of Sport, No. 11DZ2261100) and the Capacity Building Plan for Local Colleges and Universities of Shanghai Science and Technology Commission (No. 20080502700).

## Conflict of Interest

The authors declare that the research was conducted in the absence of any commercial or financial relationships that could be construed as a potential conflict of interest.

## Publisher's Note

All claims expressed in this article are solely those of the authors and do not necessarily represent those of their affiliated organizations, or those of the publisher, the editors and the reviewers. Any product that may be evaluated in this article, or claim that may be made by its manufacturer, is not guaranteed or endorsed by the publisher.
